# Towards sustainable urea electro-oxidation: a thermodynamic and green chemistry evaluation of alternative pathways

**DOI:** 10.1098/rsos.250156

**Published:** 2025-07-23

**Authors:** Vyacheslav Protsenko

**Affiliations:** ^1^ Department of Physical Chemistry, Ukrainian State University of Science and Technologies, Dnipro, Ukraine

**Keywords:** electrochemical hydrogen production, urea-oxidation reaction, green chemistry metrics, electricity economy, electrocatalyst development

## Abstract

This study presents a comparative thermodynamic analysis of various pathways for electrochemical hydrogen production coupled with the anodic oxidation of urea, offering a sustainable alternative to the conventional oxygen evolution reaction. For the first time, the feasibility and efficiency of these processes were evaluated using integrated green chemistry metrics, including atom economy and a newly proposed metric, electricity economy, which quantifies the theoretical minimum electrical energy required for the equilibrium formation of reaction products. The analysis demonstrated that urea-oxidation pathways generally require significantly less energy input than water electrolysis. Among the examined reactions, the oxidation of urea to gaseous nitrogen and carbonate ions was identified as the most efficient, with an electricity economy of –4650.83 J mol^–1^ and an atom economy of 6.4%. However, practical application is hindered by issues such as low product selectivity and high anodic potentials dictated by the redox thermodynamics of commonly used nickel-based catalysts. These findings underscore the need for next-generation electrocatalysts with enhanced selectivity and lower overpotentials to fully exploit the energetic advantages of urea oxidation for green hydrogen production.

## 1. Introduction

The concept of hydrogen energy, first proposed by Bockris [1], aims to prevent catastrophic climate change resulting from the large-scale combustion of fossil fuels by transitioning to hydrogen as an energy carrier [2,3]. This concept heavily relies on utilizing the water electrolysis reaction for the synthesis of green hydrogen [4–6],


(1.1) $$2H_{2}O\to 2H_{2}+O_{2}.$$

The overall reaction (1.1) is a combination of the cathodic hydrogen evolution reaction (HER) and the anodic oxygen evolution reaction (OER). For these reactions in an alkaline medium, the corresponding reaction equations are as follows:


(1.2) $$2H_{2}O\to 2e^{-}+H_{2}+2OH^{-},$$

(1.3) $$4OH^{-}\to O_{2}+2H_{2}O+4e^{-}.$$

As is well known, the theoretical decomposition voltage (the thermodynamic minimum voltage of an electrolyser) for water decomposition at a temperature of 298 K is 1.23 V, which is a relatively high value. Moreover, due to electrode polarization and ohmic losses, the total voltage of the electrolyser during water electrolysis reaches even higher levels, approximately 2.2−2.4 V [7]. Consequently, this poses economic challenges by increasing energy consumption. While ohmic losses can be fundamentally reduced through rational electrolyser design and the optimal composition of the electrolyte, and electrode polarization can be mitigated by developing and employing efficient electrocatalysts, the thermodynamic parameter of the theoretical decomposition voltage represents a natural limit. This limit is intrinsic to the reaction and, from a thermodynamic standpoint, cannot be lowered under the given temperature conditions.

A possible approach to addressing this issue is to replace the OER with an alternative anodic process that has more favourable energetics [8,9]. One of the most promising combinations involves coupling the HER with the electrochemical oxidation of urea (UOR) [10–19],


(1.4) $$CO(NH_{2}{)}_{2}(aq)+6OH^{-}\to N_{2}(g)+5H_{2}O+CO_{2}(g)+6e^{-}.$$

Alternatively, considering that in a highly alkaline environment the final product of the reaction will be carbonate ions rather than carbon dioxide, a more accurate representation of the UOR is as follows:


(1.5) $$CO(NH_{2}{)}_{2}(aq)+8OH^{-}\to N_{2}(g)+6H_{2}O+CO_{3}^{2}(aq)+6e^{-}.$$

As repeatedly indicated in the literature [10–12,20–22], the thermodynamic open-circuit voltage (OCV) for the overall reaction involving HER and UOR at 298 K is 0.37 V, which is significantly lower than 1.23 V for the combination of HER and OER in reaction (1.1).

Although the kinetics and mechanism of reaction (1.4) have been the subject of numerous and detailed studies [10–22], the thermodynamic aspects of this process are significantly less explored and the available data on this matter are somewhat contradictory. Specifically, in our previous works, it was established that the thermodynamic OCV for the overall reaction involving HER and UOR at 298 K, excluding carbonate formation, is not 0.37 V but only 0.072 V [23]. When accounting for the inevitable formation of carbonates in an alkaline environment (reaction (1.5)), which is commonly used for water electrolysis, the overall reaction becomes thermodynamically spontaneous with an electromotive force of 0.024 V [24]. These results, on the one hand, further emphasize the great potential of using UOR to replace OER in green hydrogen synthesis and on the other hand, highlight the insufficient understanding of the thermodynamic aspects of electrochemical reaction (1.4), which clearly hinders further progress in this scientific field.

Moreover, the literature has noted that reaction (1.4) is not the only possible anodic electrochemical transformation of urea at the anode in an alkaline environment, and the following alternative oxidation processes may occur [25–27]. Specifically, the following alternative processes are considered^1^ [28]:


(1.6) $$CO(NH_{2}{)}_{2}(aq)+8OH^{-}(aq)\to OCN^{-}(aq)+6H_{2}O(l)+NO_{2}^{-}(aq)+6e^{-},$$

(1.7) $$CO(NH_{2}{)}_{2}(aq)+10OH^{-}(aq)\to N_{2}O(g)+7H_{2}O(l)+CO_{3}^{2-}(aq)+8e^{-},$$

(1.8) $$CO(NH_{2}{)}_{2}(aq)+16OH^{-}(aq)\to 2NO_{2}^{-}(aq)+10H_{2}O(l)+CO_{3}^{2-}(aq)+12e^{-}$$

and


(1.9) $$CO(NH_{2}{)}_{2}(aq)+20OH^{-}(aq)\to 2NO_{3}^{-}(aq)+12H_{2}O(l)+CO_{3}^{2-}(aq)+16e^{-}.$$

Although reference [28] provides Gibbs free energy changes and standard potentials for these competing reactions, a comprehensive thermodynamic treatment including standard enthalpy and entropy changes remains absent in the literature, yet is crucial for uncovering mechanistic subtleties and evaluating the true energy efficiency of UOR for green hydrogen production.

It should be observed that the processes of CO(NH_2_)_2_ electrochemical oxidation are of significant importance and relevance not only for hydrogen synthesis in renewable energy and the development of so-called direct urea fuel cells [29–32]. Since urea is produced in large quantities as a result of animal and human metabolism and inevitably ends up in domestic and industrial wastewater, as well as wastewater from agro-industrial enterprises, the urgent issue of urea removal from wastewater arises. This can, among other things, be successfully addressed using electrochemical purification methods based on UOR [33–35].

Since the concurrent processes of anodic urea oxidation are an obvious subject of consideration within the framework of green chemistry concepts [36–39], their evaluation in light of key green chemistry metrics [40–43] is of significant interest. In particular, in this work, we have calculated and discussed the quantitative parameters characterizing atom economy and design for energy efficiency for various pathways of anodic urea degradation in accordance with the second and sixth principles of green chemistry^2^ [36].

Green chemistry principles have long been successfully implemented in electrochemical processes [44–48], leading to the development of technologies in which the use or generation of substances harmful to the environment and to operating personnel is greatly reduced or entirely eliminated. It is, therefore, appropriate to apply standard green chemistry principles and common sustainability metrics, such as atom economy, energy intensity and the environmental factor (*E*-factor) [40–43], to electrochemical systems. However, a survey of the literature, including recent reviews, shows that no eco-efficiency metrics have been developed or implemented that account for the unique nature and mechanisms of electrochemical reactions involving the conversion between electrical and chemical energy [40,41,43–50]. Without such specialized metrics, a comprehensive comparative analysis of the various anodic urea-oxidation pathways within a green chemistry framework cannot be achieved, particularly when evaluating and comparing the energy requirements of electrode processes. The development and implementation of green metrics for energy efficiency that reflect the specific characteristics of electrochemical reactions is, therefore, both important and timely.

Considering the aforementioned relevant issues, the aim of this study is a comprehensive analysis of the anodic urea-oxidation processes, taking into account the thermodynamic parameters of various competing pathways of this electrochemical reaction. The study also focuses on evaluating the alignment of these processes with the criteria of atom economy and energy efficiency in the context of the principles of green chemistry.

## 2. Calculations

The thermodynamic characteristics of the reactions were calculated based on tabulated values of standard enthalpies of formation and standard entropies for all molecules and ions involved in the considered reactions (electronic supplementary material, table S1). The conversion of the standard enthalpy of formation and entropy of urea from its solid state to the dissolved state was carried out using literature data [51] on the values of the enthalpy and entropy of urea dissolution, Δ*H* = 16.91 kJ mol^–1^ and Δ*S* = 62.59 J mol^–1^ K^–1^, according to the algorithm previously described [23,24].

All calculations were performed at a temperature of 298 K. The standard enthalpy of reactions (Δ*H*^0^_298_) and the entropy changes (Δ*S*^0^_298_) were determined as the difference between the total product and total reactant molar enthalpies and entropies, respectively. The standard Gibbs free energy change (Δ*G*^0^_298_) was calculated using the Gibbs–Helmholtz equation,


(2.1) $$\Delta G_{298}^{0}=\Delta H_{298}^{0}-298\Delta S_{298}^{0}.$$

The thermodynamic OCV for the electrochemical reactions was calculated using the following formula:


(2.2) $$OCV=\frac{\Delta G_{298}^{0}}{zF},$$

where *z* is the number of electrons involved in the reaction under consideration, and *F* is the Faraday constant, 96 485 С mol^–1^.

Atom economy was calculated using the equation [52]


(2.3) $$atom\;economy=⟮\frac{m.w.\text{of the product}}{m.w.\text{of the reagents}}⟯\times 100\%,$$

where the numerator includes the molecular weight of the product, and the denominator contains the total molecular weights of all the starting reagents.

The *E-*factor was calculated using the equation [41,43]


(2.4) $$E\text{-factor}=\frac{m.w.\text{of wastes}}{m.w.\text{of the product}},$$

where the numerator contains the molecular weights of the produced wastes, and the denominator contains the molecular weight of the desired target product produced in the stoichiometric equation.

## 3. Results and discussion

The calculated values of standard enthalpy changes, entropy changes, Gibbs free energy, OCV, atom economy and *E*-factor for various reaction pathways are summarized in table 1. This table presents data for the water electrolysis reaction (reaction (I)) as well as for the balanced overall electrochemical reactions composed of anodic half-reactions (1.4–1.9) and the cathodic half-reaction (1.2), (reactions (II)–(VII)).

**Table 1.** Calculated thermodynamic parameters and green chemistry metrics for the reactions under consideration.

reaction number	reaction	Δ*H*^0^_298_ (kJ mol^–1^)	Δ*S*^0^_298_ (J mol^–1^ K^–1^)	Δ*G*^0^_298_ (J mol^–1^)	OCV (V)	atom economy (%)	*E-*factor
I	$H_{2}O(l)\pm 2e^{-}\to H_{2}(g)+0.5O_{2}(g)$	285.83	163.09	237229.18	1.229	11.1	0
II	$\begin{array}{r} CO(NH_{2}{)}_{2}(aq)+H_{2}O(l)\pm 6e^{-}\to \\ \to CO_{2}(g)+N_{2}(g)+3H_{2}(g) \end{array}$	208.58	559.58	41825.16	0.072	7.7	7.3
III	$\begin{array}{r} CO(NH_{2}{)}_{2}(aq)+2OH^{-}(aq)\pm 6e^{-}\to \\ \to CO_{3}^{2-}(aq)+N_{2}(g)+3H_{2}(g) \end{array}$	99.66	381.25	−13952.50	−0.024	6.4	10.0
IV	$\begin{array}{r} CO(NH_{2}{)}_{2}(aq)+2OH^{-}(aq)\pm 6e^{-}\to \\ \to OCN^{-}(aq)+NO_{2}^{-}(g)+3H_{2}(g) \end{array}$	525.70	475.49	384003.98	0.663	6.4	7.0
V	$\begin{array}{r} CO(NH_{2}{)}_{2}(aq)+2OH^{-}(aq)+H_{2}O(l)\pm 8e^{-}\to \\ \to N_{2}O(aq)+CO_{3}^{2-}(aq)+4H_{2}(g) \end{array}$	467.09	470.32	326934.64	0.424	7.1	13.0
VI	$\begin{array}{r} CO(NH_{2}{)}_{2}(aq)+4OH^{-}(aq)+2H_{2}O(l)\pm 12e^{-}\to \\ \to 2NO_{2}^{-}(aq)+CO_{3}^{2-}(aq)+6H_{2}(g) \end{array}$	922.16	708.83	710928.66	0.614	7.3	12.7
VII	$\begin{array}{r} CO(NH_{2}{)}_{2}(aq)+4OH^{-}(aq)+4H_{2}O(l)\pm 16e^{-}\to \\ \to 2NO_{3}^{-}(aq)+CO_{3}^{2-}(aq)+8H_{2}(g) \end{array}$	1288.22	876.77	1026942.54	0.665	8.0	11.5

It is evident that all these electrochemical processes are endothermic, which is largely determined by the fact that the reactants involved have very negative values of formation enthalpy. Specifically, this applies to liquid water, dissolved urea and the hydroxide ion, which also has a very negative formation enthalpy. Therefore, the consumption of these reagents, which possess sufficiently high bond energies, requires the absorption of heat, which dictates the overall endothermic nature of the processes. A particularly large amount of heat is absorbed in reaction (VI) with the formation of nitrate ions as one of the final products. When comparing the thermal effects of reactions (II)–(VII) with the value characteristic of water electrolysis (I), it is observed that for reactions producing gaseous nitrogen, the heat of the process is lower than in reaction (I), while for reactions that involve the formation of other nitrogen oxidation products, it is significantly higher.

However, the determined amounts of heat, as is typically the case in thermochemical calculations, depend on the stoichiometric equation of the reaction. Essentially, these heats are referenced to different quantities of synthesized hydrogen, which is the target product in all these processes. If we proceed to list the thermodynamic parameters, specifically the isobaric heat of reaction calculated per mole of H_2_, the situation becomes somewhat different (table 2). In such a comparison, the amount of heat absorbed in all reactions involving urea is significantly lower than in water electrolysis. The least amount of heat is absorbed in the reaction that involves the formation of gaseous nitrogen and carbonate ions as the final products of urea decomposition (reaction (III)).

**Table 2.** Calculated changes in standard enthalpy, entropy and Gibbs energies converted to one mole of produced hydrogen.

reaction number	reaction	Δ*H*^0^_298_ (kJ)	Δ*S*^0^_298_ (J K^–1^)	Δ*G*^0^_298_ (J)
I	$H_{2}O(l)\pm 2e^{-}\to H_{2}(g)+0.5O_{2}(g)$	285.83	163.09	237229.18
II	$CO(NH_{2}{)}_{2}(aq)+H_{2}O(l)\pm 6e^{-}\to CO_{2}(g)+N_{2}(g)+3H_{2}(g)$	69.53	186.53	13941.72
III	$CO(NH_{2}{)}_{2}(aq)+2OH^{-}(aq)\pm 6e^{-}\to CO_{3}^{2-}(aq)+N_{2}(g)+3H_{2}(g)$	33.22	127.08	−4650.83
IV	$CO(NH_{2}{)}_{2}(aq)+2OH^{-}(aq)\pm 6e^{-}\to OCN^{-}(aq)+NO_{2}^{-}(aq)+3H_{2}(g)$	175.23	158.50	128001.33
V	$CO(NH_{2}{)}_{2}(aq)+2OH^{-}(aq)+H_{2}O(l)\pm 8e^{-}\to N_{2}O(g{)}^{-}+CO_{3}^{2-}(aq)+4H_{2}(g)$	116.77	117.58	81733.66
VI	$CO(NH_{2}{)}_{2}(aq)+4OH^{-}(aq)+H_{2}O(l)\pm 12e^{-}\to 2NO_{2}^{-}(aq)+CO_{3}^{2-}(aq)+6H_{2}(g)$	153.69	118.14	118488.11
VII	$CO(NH_{2}{)}_{2}(aq)+4OH^{-}(aq)+4H_{2}O(l)\pm 16e^{-}\to 2NO_{3}^{-}(aq)+CO_{3}^{2-}(aq)+8H_{2}(g)$	161.03	109.60	128367.82

All the electrochemical transformations considered (I)–(VII) are accompanied by an increase in entropy (table 1). This result is due to the formation of a large number of gases (hydrogen, nitrogen, nitrous oxide and carbon dioxide) during these reactions, which, as is known, have high molar entropies compared with liquids and solids. The increase in entropy from a thermodynamic perspective signifies an increase in disorder and a degradation of energy. From fundamental ecological considerations, the increase in entropy in the anthroposphere of our planet is an undesirable effect. Indeed, minimizing entropy production should be considered one of the criteria for sustainability [53]. However, it should be noted that hydrogen, produced in the reactions under consideration, is intended for future use as an environmentally safe fuel, for example through its oxidation in fuel cells via a reaction reverse to reaction (I), which is obviously accompanied by a corresponding decrease in entropy. Thus, the overall entropy of Earth as a thermodynamic system will not increase significantly. Only thermodynamically irreversible processes, such as heat release due to ohmic losses in electrolysers and fuel cells and due to polarization, will lead to irreversible increases in the planet’s entropy. However, these processes are not the subject of detailed consideration in this publication. Interestingly, the recalculation of entropy change produced per mole of hydrogen in different reactions shows (table 2) that the differences between these values for reactions (I)–(VII) are largely eliminated. This means that the main factor determining the increase in entropy in these processes, at first approximation, is the formation of gaseous hydrogen.

The standard changes in Gibbs energy during the considered reactions are positive, which is determined by the positive enthalpy factors that prevail over the also positive entropy factors. From a thermodynamic perspective, positive values of Δ*G*^0^_298_ indicate that these processes are non-spontaneous and require energy input from external sources (which is quite natural for non-spontaneous reactions during electrolysis). The only exception to this is reaction (III), with the formation of nitrogen and carbonate ions as products of urea decomposition, for which Δ*G*^0^_298_ < 0 [24]. This is probably due to the relatively low standard enthalpy change for this reaction, which cannot compensate for the positive entropy factor in the Gibbs–Helmholtz equation. Therefore, such a process would occur spontaneously, for example, in a galvanic cell, as indicated by the negative sign for the OCV of this reaction (table 1). However, as is known, this process does not actually occur spontaneously but is realized non-spontaneously during electrolysis, and moreover, it requires a relatively high decomposition voltage, although lower than for water splitting (I) [10–12]. The reason for this is the very slow kinetics of the process [24].

Based on the calculated OCV values and taking into account the standard potential of half-reaction (1.2) (−0.828 V versus the standard hydrogen electrode), the standard potentials of half-reactions (1.6–1.9) were calculated (electronic supplementary material, table S2). The obtained values are virtually identical within the acceptable calculation error to the values reported in the study [28], except for half-reaction (1.6), for which we obtained a value of −0.165 V, while the mentioned work reported a value of −0.43 V. The reasons for such a significant discrepancy are currently unclear.

The OCV values, which were obtained from the calculated Δ*G*^0^_298_, for any of the considered reaction pathways of electrochemical hydrogen synthesis with the coupled urea-oxidation half-reaction (II)–(VII) are lower than 1.299 V, which is characteristic of water splitting, thus highlighting the exceptional potential of this approach for green hydrogen energy.

However, not all of these urea-involved processes are equally environmentally attractive, as they are accompanied by the formation of various waste products of different quantities and levels of environmental hazard. For a quantitative assessment of this hazard, we used well-known metrics from green chemistry theory and practice: atom economy and the environmental factor (*E*-factor) (table 1).

Atom economy essentially characterizes the degree to which all materials used in the process are incorporated into the final product. For processes that are ideally clean from the perspective of sustainable chemistry, this value is 100%. The lower the atom economy, the larger the relative amount of starting materials that are not converted into the final product but instead into waste that must be disposed of or recycled. It is evident that all the reactions discussed, including the conventional water electrolysis reaction (I), show relatively low atom economy values of 6−11%. These relatively low values of the metric are a consequence of the specific and unique small molecular weight of hydrogen, which is considered the final product here. Therefore, such low values do not necessarily indicate the low environmental attractiveness of hydrogen electrolysis processes. One must take into account the environmental hazard of the by-products formed along with hydrogen. Hence, in this case, a more accurate approach would be the use of the *E*-factor proposed by Sheldon [39,41], which assesses the amount of hazardous waste per unit mass of the substance that is the final product of chemical synthesis.

The calculated *E*-factor values, considering theoretical stoichiometry, are presented in table 1. In these calculations, it was taken into account that some of the products formed in the reactions under consideration are environmentally safe (gaseous nitrogen and oxygen, even if they are directly released into the atmosphere). Therefore, in calculating the *E*-factor, these were excluded, just as water is typically excluded in such calculations. Carbonate ions (e.g. in the form of alkali metal salts), although not highly harmful or toxic, will require periodic removal from the solutions used in electrolysis (e.g. through chemical treatment [54]), which will inevitably lead to the formation of new waste that will need to be recovered. Therefore, carbonate ions were included in the calculations as waste. Finally, nitrous oxide (N_2_O), as well as cyanate, nitrate and nitrite ions, are undoubtedly harmful environmental pollutants [55–58] and were also considered as waste in our calculations.

The smaller the *E*-factor, the more environmentally safe the process is. From the results of the calculations, it is evident that the most environmentally clean process is water splitting (reaction (I)), as it does not produce any harmful emissions. However, it should be considered that the values we obtained are theoretical and do not account for emissions in auxiliary processes and technologies. Specifically, it should be taken into account that in alkaline water electrolysis processes, sufficiently concentrated alkaline solutions are used, which need to be periodically purified and regenerated, leading to the formation of some waste, which was obviously not considered in our calculations. A cleaner version of reaction (I) would be its implementation in proton exchange membrane water electrolysers, where pure water, not a concentrated alkaline solution, is used as the starting reagent [59]. However, even in these processes, throughout the full life cycle of the electrolysers and ion-exchange membranes used, a certain amount of waste will be generated.

The highest *E*-factor values are obtained for processes that lead to the formation of toxic nitrogen compounds derived from urea molecules: N_2_O and nitrite and nitrate ions. These are undesirable pathways for the process and should be avoided by developing highly selective electrocatalysts that will drive the process through alternative routes.

One of the smallest *E*-factor values is associated with reaction (II) (table 1), where the products of urea conversion are carbon dioxide and molecular nitrogen. However, the relative ecological safety of this route is somewhat illusory, as the emission of greenhouse gas CO_2_ directly contradicts the concept of creating carbon-free renewable energy. Nevertheless, in our opinion, the practical implementation of this reaction pathway is unlikely, as carbon dioxide will quickly be absorbed in the concentrated alkaline solution for electrolysis and process (III) will probably occur, which involves the formation of carbonate ions as the final products of the reaction. This, of course, changes the thermodynamic parameters of the reaction, which are state-dependent [24].

Thus, the analysis of atomic efficiency and *E*-factor values indicates that the most effective alternative to the OER in water electrolysis is reaction (III). The same conclusion follows from the application of the energy efficiency principle, which also plays an important role in analysing alternative options from the perspective of green chemistry principles. Indeed, as shown above, reaction (III), which involves the formation of carbonate ions and molecular nitrogen, is the only one that is thermodynamically spontaneous and should proceed without the consumption of external electrical energy, unlike all other reactions. On the contrary, it would generate electrical energy, following the principle of a galvanic cell.

In this context, it should be emphasized that for the analysis of the energy efficiency of an electrochemical reaction within the framework of the green chemistry approach, comparing the values of standard electrochemical potentials is not flawless. The reason is that different numbers of electrons are involved in the reactions being considered, which directly affects energy consumption and must also be taken into account during the analysis. However, the value of the standard potential does not account for this feature and says nothing about these differences. Therefore, a more accurate metric of energy efficiency should be the change in the standard Gibbs energy during the reaction, calculated per unit of substance (or mass) of the final product,


(3.1) $$electricity\;economy=\frac{zF\cdot OCV}{n}=\frac{\Delta G}{n},$$

where *z* is the number of electrons in the overall electrochemical reaction equation; *F* is the Faraday constant; OCV is the thermodynamic open-circuit voltage; and *n* is the stoichiometric coefficient of the final product in the reaction equation being considered.

This value is analogous to the atomic economy metric and can be conditionally referred to as the *electricity economy*. In essence, for thermodynamically non-spontaneous electrochemical reactions (Δ*G *> 0), this quantity represents the minimum amount of external electrical energy required for the equilibrium formation of 1 mole (or 1 kg) of the final product of the reaction. The smaller the value of the electricity economy, the lower the minimum possible energy expenditure for the target electrochemical reaction to proceed, and consequently, the more efficient the process is in the context of the development of green chemistry.

It is clear that the electricity economy is a purely theoretical quantity that only allows for an estimation of the lower bound of potential energy consumption. In real electrochemical processes, energy consumption will be higher due to electrode polarization and losses through ohmic components. Moreover, total electrical energy consumption can increase due to a reduction in the current efficiency of the target reaction. However, theoretically, electrode reaction polarization can be reduced by selecting highly efficient electrocatalysts, and ohmic components can be minimized through rational equipment design and variation in the composition and concentration of electrolytes. The thermodynamic quantity of electricity economy, however, is an unchanging attribute of each specific electrochemical reaction under given conditions and thus can be considered as another fundamental metric for analysis in green chemistry.

It is noteworthy that for thermodynamically spontaneous electrochemical processes (Δ*G *< 0), the value of the electricity economy takes on a conditional negative sign and resembles the well-known metric in the field of chemical power sources—the energy density of a power source. The higher this value in absolute terms, the better the power source (battery or accumulator) is in this regard.

**Figure 1.**
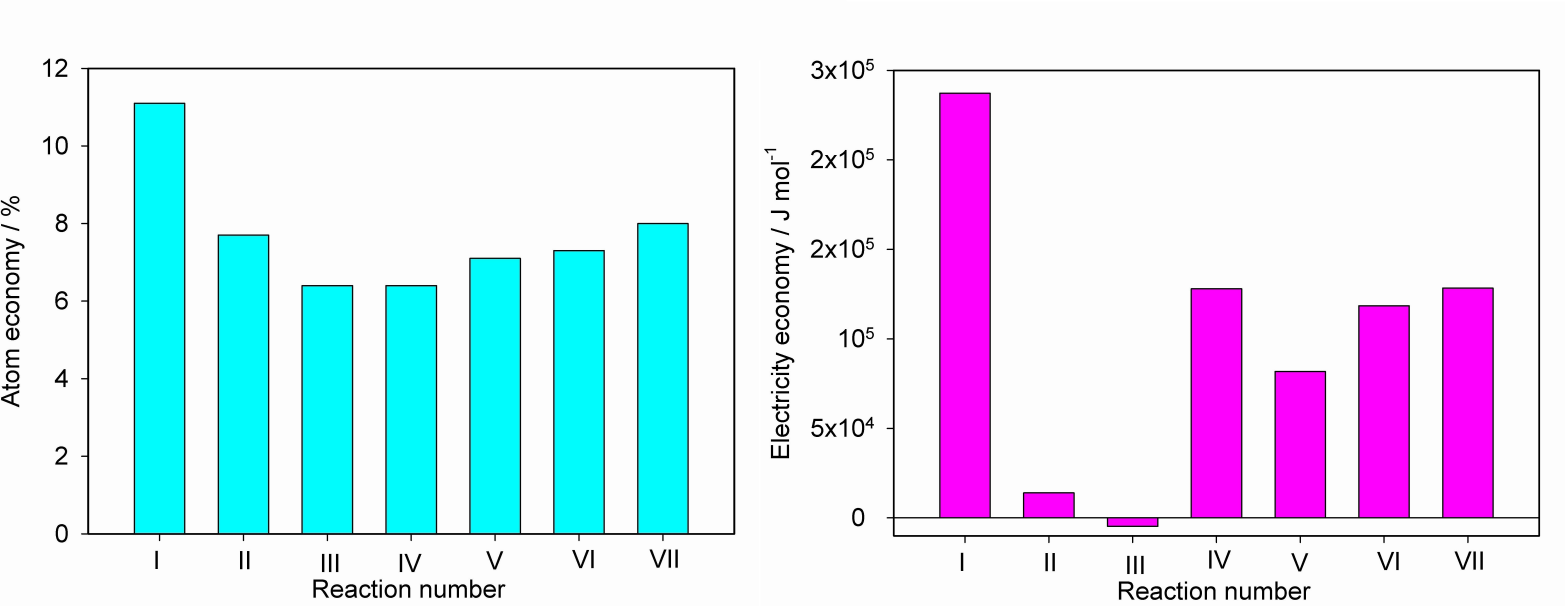
Bar charts comparing atom economy and electricity economy metrics for seven reaction pathways under consideration.

A comparison of the calculated electricity economy values for the hydrogen electrosynthesis reactions (I)–(VII) (the last column in table 2) shows that in all alternative reaction pathways involving urea (reactions (II)–(VII)), the electricity economy is lower than for conventional water electrolysis (reaction (I)) (figure 1). The most advantageous, obviously, is reaction (III) (a thermodynamically spontaneous process), with reaction (II) producing gaseous nitrogen and carbon dioxide coming in second. Reactions involving the formation of N_2_O and nitrite, nitrate and cyanate ions are less attractive in terms of energy efficiency.

From the data presented in figure 1, it is evident that selecting the most environmentally friendly alternative among the seven reaction pathways under consideration should be based on an analysis of the electricity economy metric. Indeed, all the reactions exhibit very similar atom economy values, whereas their electricity economy metrics differ significantly, making it the decisive factor for evaluating potential environmental attractiveness.

## 4. Conclusions and future directions

(1) For the first time, a comparison and analysis of various pathways for the electrochemical synthesis of hydrogen combined with the anodic electro-oxidation of urea as an alternative to the oxygen evolution reaction have been conducted. This study represents the first systematic comparison of different hydrogen synthesis routes involving urea oxidation, considered a potential alternative to the traditional OER. The analysis accounted for both thermodynamic aspects and green chemistry metrics, such as atom economy, *E*-factor and energy efficiency. This comprehensive evaluation highlighted the advantages and limitations of each process in terms of environmental safety, energy consumption and integration potential into sustainable energy systems. Using urea as a feedstock for green hydrogen production is not only chemically intriguing but also opens new possibilities for developing eco-friendly and energy-efficient technologies.
(2) The feasibility and efficiency of replacing the OER with the urea oxidation reaction within the framework of green chemistry concepts have been demonstrated. A comparison of reactions involving UOR instead of the conventional OER revealed significant advantages in the context of green chemistry. This approach not only reduces energy consumption during hydrogen production processes but also minimizes the formation of harmful by-products. Reactions involving urea generate less toxic and more manageable by-products, such as gaseous nitrogen and carbonate ions, making the process more environmentally friendly. Moreover, the use of urea, an accessible and inexpensive raw material compared with water, offers scalability for industrial applications. This approach significantly contributes to the advancement of sustainable hydrogen energy technologies, which are crucial for the green energy revolution.
(3) A new metric, electricity economy, has been proposed for evaluating energy efficiency. Its meaning and significance have been analysed. Reactions involving urea have been assessed in light of this new metric. One of the key outcomes of this study is the introduction of this new metric, electricity economy, to assess the energy efficiency of electrochemical processes. This metric evaluates the minimum required amount of electrical energy for each reaction, taking into account the thermodynamic spontaneity of the process. Calculations showed that urea-based processes exhibit significantly better efficiency compared with traditional water electrolysis, mainly due to the thermodynamic spontaneity of a certain reaction. Assessing reactions using the electricity economy metric provides a clearer understanding of the potential for reducing energy consumption and increasing overall energy efficiency, which is critically important for the further development of renewable energy technologies.
  The electricity economy metric introduced in this study offers a theoretical baseline for assessing the minimum electrical energy input required per mole of hydrogen produced in urea-assisted electrolysis. While it captures the thermodynamic ideal, its relationship to practical performance indicators, such as faradaic efficiency or energy recovery rate, remains to be established. Future work will focus on bridging this gap by applying the electricity economy concept to experimental systems or techno-economic simulations. Such integration would provide a more comprehensive picture of how closely real-world processes approach the thermodynamic limit.
(4) The analysis shows that the most efficient reaction from the perspective of green chemistry is the urea oxidation reaction that leads to the formation of carbonate ions and gaseous nitrogen. This process is thermodynamically spontaneous, which helps reduce the energy required for its implementation. However, as evidenced by literature data [10,11], first, the process is not always selective, and other nitrogen oxidation products harmful to the environment may form. Second, the electrode potentials at which urea oxidation occurs at the anode are generally determined not by the thermodynamic characteristics of the reaction itself but by the thermodynamics of the Ni(III)/Ni(II) redox couple, which is the most widely used electrocatalyst. These potentials, although lower than for the OER, are still relatively high.
  This highlights an important future task: the development of new electrocatalysts for the urea oxidation reaction that would be highly selective and efficient. In this context, the search for alternative electrocatalyst materials beyond nickel-based systems becomes essential. Future studies should explore catalyst classes capable of modulating reaction pathways with greater precision, such as transition metal alloys, doped carbons, molecular catalysts and other advanced materials, and investigate their ability to reduce onset potentials and improve product selectivity in urea electro-oxidation. This would fully realize the significant thermodynamic potential embedded in the urea oxidation reaction. Innovations in this area of electrochemistry will contribute to the development of sustainable, economically viable and environmentally friendly hydrogen production technologies.
(5) It is important to recognize that, in real electrochemical systems, urea oxidation pathways may compete with simultaneous oxygen evolution, since electrocatalysts rarely achieve perfect selectivity. From the standpoint of atom economy and *E*‑factor, the presence of oxygen alongside gaseous nitrogen does not diminish the green‑chemistry profile, as both gases can be safely released to the atmosphere. Regarding energy consumption, if the anodic potential remains governed primarily by urea‑oxidation kinetics and OER occurs only as a side reaction, the electrical charge required per mole of H_2_ is unchanged (2 F per mole, regardless of whether OER is involved). These considerations suggest that future catalyst development should prioritize selectivity among urea‑oxidation pathways, while competing oxygen evolution plays a secondary role in overall sustainability performance.
(6) It should be noted that a promising extension of the present work is the application of life‑cycle costing (LCC) and life‑cycle assessment (LCA) methodologies [60]. LCC provides a comprehensive estimation of economic expenditures associated with a process, including capital investment, operational expenses, maintenance and end‑of‑life disposal, thereby revealing the true cost efficiency of each urea oxidation pathway over its entire service life. Similarly, LCA systematically quantifies material and energy inputs, emissions and resource depletion from cradle to grave, offering a detailed assessment of the environmental footprint for each reaction route. Integrating thermodynamic performance metrics with LCC and LCA data would enable identification of trade‑offs among energy efficiency, economic viability and ecological impact, thus guiding the design and selection of the most sustainable electrocatalytic systems. However, these multidisciplinary approaches extend beyond the classical thermodynamic framework and green‑chemistry metrics employed in this study. Their full implementation lies outside the stated objectives, but they represent a valuable and attractive direction for future research.
(7) While this study is theoretical in nature and based on thermodynamic principles and sustainability metrics, future work will focus on experimental validation of the most promising urea oxidation pathways identified herein. Such studies will involve the measurement of cell voltage, faradaic efficiency, and reaction rates under controlled electrochemical conditions, in order to verify and refine the thermodynamic predictions and assess the real-world feasibility of the proposed processes.

## Data Availability

The datasets supporting this article have been uploaded as part of the electronic supplementary material [61]. Supplementary material is available online [62].

## Footnotes

^1^
Several hypothetical reactions, in which gaseous CO_2_ and HCO_3_^-^ ions are formed, are not presented here, as this is unlikely to occur in strongly alkaline environments typically used in water electrolysis.

^2^
These principles are formulated as follows: ‘2. Synthetic methods should be designed to maximize the incorporation of all materials used in the process into the final product... 6. Energy requirements should be recognized for their environmental and economic impacts and should be minimized...’ [36].

## References

[1] Bockris JO'M. 2022 The origin of ideas on a hydrogen economy and its solution to the decay of the environment. Int. J. Hydrog. Energy **27**, 731–740. (10.1016/S0360-3199(01)00154-9)

[2] Bogdanov D *et al*. 2021 Low-cost renewable electricity as the key driver of the global energy transition towards sustainability. Energy **227**, 120467. (10.1016/j.energy.2021.120467)

[3] Squadrito G, Maggio G, Nicita A. 2023 The green hydrogen revolution. Renew. Energy **216**, 119041. (10.1016/j.renene.2023.119041)

[4] Shih AJ *et al*. 2022 Water electrolysis. Nat. Rev. Methods Prim. **2**, 84. (10.1038/s43586-022-00164-0)

[5] Dawood F, Anda M, Shafiullah GM. 2020 Hydrogen production for energy: an overview. Int. J. Hydrog. Energy **45**, 3847–3869. (10.1016/j.ijhydene.2019.12.059)

[6] Ajanovic A, Sayer M, Haas R. 2022 The economics and the environmental benignity of different colors of hydrogen. Int. J. Hydrog. Energy **47**, 24136. (10.1016/j.ijhydene.2022.02.094)

[7] Xiang C, Papadantonakis KM, Lewis NS. 2016 Principles and implementations of electrolysis systems for water splitting. Mater. Horizons **3**, 169–173. (10.1039/c6mh00016a)

[8] Wang C, Wu Y, Bodach A, Krebs ML, Schuhmann W, Schüth F. 2023 A novel electrode for value‐generating anode reactions in water electrolyzers at industrial current densities. Angew. Chem. Int. Ed. **62**, e202215804. (10.1002/anie.202215804) PMC10107951 36440966

[9] Zhou W, Chen S, Meng X, Li J, Gao J. 2023 Energy-saving cathodic H_2_ production enabled by non-oxygen evolution anodic reactions: a critical review on fundamental principles and applications. Int. J. Hydrog. Energy **48**, 15748–15770. (10.1016/j.ijhydene.2023.01.063)

[10] Paygozar S, Aghdam ASR, Hassanizadeh E, Andaveh R, Darband GB. 2023 Recent progress in non-noble metal-based electrocatalysts for urea-assisted electrochemical hydrogen production. Int. J. Hydrog. Energy **48**, 7219–7259. (10.1016/j.ijhydene.2022.11.087)

[11] Anuratha K, Rinawati M, Wu TH, Yeh MH, Lin JY. 2022 Recent development of nickel-based electrocatalysts for urea electrolysis in alkaline solution. Nanomaterials **12**, 2970. (10.3390/nano12172970) 36080007 PMC9457967

[12] Boggs BK, King RL, Botte GG. 2009 Urea electrolysis: direct hydrogen production from urine. Chem. Commun. (Camb.) **2009**, 4859–4861. (10.1039/b905974a) 19652805

[13] Vedharathinam V, Botte GG. 2012 Understanding the electro-catalytic oxidation mechanism of urea on nickel electrodes in alkaline medium. Electrochim. Acta **81**, 292–300. (10.1016/j.electacta.2012.07.007)

[14] Wang D, Yan W, Vijapur SH, Botte GG. 2012 Enhanced electrocatalytic oxidation of urea based on nickel hydroxide nanoribbons. J. Power Sources **217**, 498–502. (10.1016/j.jpowsour.2012.06.029)

[15] Li J, Zhang J, Yang JH. 2022 Research progress and applications of nickel-based catalysts for electrooxidation of urea. Int. J. Hydrog. Energy **47**, 7693–7712. (10.1016/j.ijhydene.2021.12.099)

[16] Lu S, Zheng X, Fang L, Yin F, Liu H. 2023 Rational engineering design of nickel hydroxides for urea oxidation reaction: a mini-review. Electrochem. Commun. **157**, 107599. (10.1016/j.elecom.2023.107599)

[17] Ge J, Liu Z, Guan M, Kuang J, Xiao Y, Yang Y, Tsang CH, Lu X, Yang C. 2022 Investigation of the electrocatalytic mechanisms of urea oxidation reaction on the surface of transition metal oxides. J. Colloid Interface Sci. **620**, 442–453. (10.1016/j.jcis.2022.03.152) 35439695

[18] Yan X, Zhang WD, Hu QT, Liu J, Li T, Liu Y, Gu ZG. 2019 Defects-rich nickel nanoparticles grown on nickel foam as integrated electrodes for electrocatalytic oxidation of urea. Int. J. Hydrog. Energy **44**, 27664–27670. (10.1016/j.ijhydene.2019.09.004)

[19] Ma Y, Ma C, Wang Y, Wang K. 2022 Advanced nickel-based catalysts for urea oxidation reaction: challenges and developments. Catalysts **12**, 337. (10.3390/catal12030337)

[20] Liu W, Qin Z, Dai X, Meng S, Niu X, Shi W, Wu F, Cao X. 2023 Coupling of NiFe layered double hydroxides with sulfides for highly efficient urea electrolysis and hydrogen evolution. Energies **16**, 1092. (10.3390/en16031092)

[21] Miller AT, Hassler BL, Botte GG. 2012 Rhodium electrodeposition on nickel electrodes used for urea electrolysis. J. Appl. Electrochem. **42**, 925–934. (10.1007/s10800-012-0478-1)

[22] Yan W, Wang D, Botte GG. 2012 Nickel and cobalt bimetallic hydroxide catalysts for urea electro-oxidation. Electrochim. Acta **61**, 25–30. (10.1016/j.electacta.2011.11.044)

[23] Protsenko VS. 2023 Thermodynamic aspects of urea oxidation reaction in the context of hydrogen production by electrolysis. Int. J. Hydrog. Energy **48**, 24207–24211. (10.1016/j.ijhydene.2023.03.295)

[24] Protsenko VS, Bobrova LS, Butyrina TE, Sukhatskyi OD. 2024 Thermodynamics of electrochemical urea oxidation reaction coupled with cathodic hydrogen evolution reaction in an alkaline solution: effect of carbonate formation. Int. J. Hydrog. Energy **59**, 354–358. (10.1016/j.ijhydene.2024.02.006)

[25] Li J *et al*. 2021 Deciphering and suppressing over‐oxidized nitrogen in nickel‐catalyzed urea electrolysis. Angew. Chem. Int. Ed. **60**, 26656–26662. (10.1002/anie.202107886) 34553818

[26] Tatarchuk SW, Medvedev JJ, Li F, Tobolovskaya Y, Klinkova A. 2022 Nickel‐catalyzed urea electrolysis: from nitrite and cyanate as major products to nitrogen evolution. Angew. Chem. Int. Ed. **61**, e202209839. (10.1002/anie.202209839) 35931655

[27] Hopsort G, Pereira Do Carmo D, Latapie L, Loubière K, Serrano KG, Tzedakis T. 2023 Progress toward a better understanding of the urea oxidation by electromediation of Ni(III)/Ni(II) system in alkaline media. Electrochim. Acta **442**, 141898. (10.1016/j.electacta.2023.141898)

[28] Akkari S *et al*. 2025 Progress on electrochemical and photoelectrochemical urea and ammonia conversion from urine for sustainable wastewater treatment. Appl. Catal. B Env. Energy **362**, 124718. (10.1016/j.apcatb.2024.124718)

[29] Gnana G, Farithkhan A, Manthiram A. 2020 Direct urea fuel cells: recent progress and critical challenges of urea oxidation electrocatalysis. Adv Energy Sustain. Res **1**, 2000015. (10.1002/aesr.202000015)

[30] Sayed ET, Eisa T, Mohamed HO, Abdelkareem MA, Allagui A, Alawadhi H, Chae KJ. 2019 Direct urea fuel cells: challenges and opportunities. J. Power Sources **417**, 159–175. (10.1016/j.jpowsour.2018.12.024)

[31] Ye K, Wang G, Cao D, Wang G. 2018 Recent advances in the electro-oxidation of urea for direct urea fuel cell and urea electrolysis. Top. Curr. Chem. **376**, 42. (10.1007/s41061-018-0219-y) 30367274

[32] Fan L, Tu Z, Chan SH. 2021 Recent development of hydrogen and fuel cell technologies: a review. Energy Rep. **7**, 8421–8446. (10.1016/j.egyr.2021.08.003)

[33] Simka W, Piotrowski J, Robak A, Nawrat G. 2009 Electrochemical treatment of aqueous solutions containing urea. J. Appl. Electrochem. **39**, 1137–1143. (10.1007/s10800-008-9771-4)

[34] Urbańczyk E, Sowa M, Simka W. 2016 Urea removal from aqueous solutions—a review. J. Appl. Electrochem. **46**, 1011–1029. (10.1007/s10800-016-0993-6)

[35] Weerakoon D, Bansal B, Padhye LP, Rachmani A, James Wright L, Silyn Roberts G, Baroutian S. 2023 A critical review on current urea removal technologies from water: an approach for pollution prevention and resource recovery. Sep. Purif. Technol. **314**, 123652. (10.1016/j.seppur.2023.123652)

[36] Anastas PT, Warner JC. 1998 Green chemistry: theory and practice. Oxford, UK: Oxford University Press.

[37] Anastas PT, Kirchhoff MM. 2002 Origins, current status, and future challenges of green chemistry. Accounts Chem. Res. **35**, 686–694. (10.1021/ar010065m) 12234198

[38] Lancaster M. 2016 Green chemistry: an introductory text. Cambridge, UK: The Royal Society of Chemistry. (10.1039/9781839168888)

[39] Sheldon RA. 2012 Fundamentals of green chemistry: efficiency in reaction design. Chem. Soc. Rev. **41**, 1437–1451. (10.1039/c1cs15219j) 22033698

[40] Constable DJC *et al*. 2001 Green chemistry measures for process research and development. Green Chem. **3**, 7–9. (10.1039/b007875l)

[41] Sheldon RA, Bode ML, Akakios SG. 2022 Metrics of green chemistry: waste minimization. Curr. Opin. Green Sustain. Chem. **33**, 100569. (10.1016/j.cogsc.2021.100569)

[42] Trost BM. 1991 The atom economy–a search for synthetic efficiency. Science **254**, 1471–1477. (10.1126/science.1962206) 1962206

[43] Martínez J, Cortés JF, Miranda R. 2022 Green chemistry metrics, a review. Processes **10**, 1274. (10.3390/pr10071274)

[44] Budnikova YH, Dolengovski EL, Tarasov MV, Gryaznova TV. 2024 Electrochemistry in organics: a powerful tool for ‘green’ synthesis. J. Solid State Electrochem. **28**, 659–676. (10.1007/s10008-023-05507-9)

[45] Ibanez JG, Fitch A, Frontana-Uribe BA, Vasquez-Medrano R. 2014 Green electrochemistry. In Encyclopedia of applied electrochemistry, pp. 964–971. New York, NY: Springer New York. (10.1007/978-1-4419-6996-5_132)

[46] Matthews MA. 2001 Green electrochemistry: examples and challenges. Pure Appl. Chem. **73**, 1305–1308. (10.1351/pac200173081305)

[47] Rajendran S, Joseph Rathish R, Santhana Prabha S, Anandanc A. 2016 Green electrochemistry – a versatile tool in green synthesis: an overview. Port. Electrochim. Acta **34**, 321–342. (10.4152/pea.201605321)

[48] Yuan Y, Lei A. 2020 Is electrosynthesis always green and advantageous compared to traditional methods? Nat. Commun. **11**, 802. (10.1038/s41467-020-14322-z) 32029716 PMC7005282

[49] Erythropel HC *et al*. 2018 The Green ChemisTREE: 20 years after taking root with the 12 principles. Green Chem. **20**, 1929–1961. (10.1039/c8gc00482j)

[50] Jimenez-Gonzalez C, Lund C. 2022 Green metrics in pharmaceutical development. Curr. Opin. Green Sustain. Chem. **33**, 100564. (10.1016/j.cogsc.2021.100564)

[51] House KA, House JE. 2017 Thermodynamics of dissolution of urea in water, alcohols, and their mixtures. J. Mol. Liq. **242**, 428–432. (10.1016/j.molliq.2017.07.020)

[52] Constable DJC, Curzons AD, Cunningham VL. 2002 Metrics to ‘green’ chemistry—which are the best? Green Chem. **4**, 521–527. (10.1039/b206169b)

[53] Addiscott TM. 1995 Entropy and sustainability. Eur. J. Soil Sci. **46**, 161–168. (10.1111/j.1365-2389.1995.tb01823.x)

[54] Sipos P, May PM, Hefter GT. 2000 Carbonate removal from concentrated hydroxide solutions. Anal. **125**, 955–958. (10.1039/a910335j)

[55] Yagiela JA. 1991 Health hazards and nitrous oxide: a time for reappraisal. Anesth. Prog. **38**, 1–991. 1809046 PMC2162364

[56] Dong K, Xie F, Chang Y, Chen C, Wang W, Lu D, Gu X. 2020 A novel strategy for the efficient decomposition of toxic sodium cyanate by hematite. Chemosphere **256**, 127047. (10.1016/j.chemosphere.2020.127047) 32446000

[57] Singh S, Anil AG, Kumar V, Kapoor D, Subramanian S, Singh J, Ramamurthy PC. 2022 Nitrates in the environment: a critical review of their distribution, sensing techniques, ecological effects and remediation. Chemosphere **287**, 131996. (10.1016/j.chemosphere.2021.131996) 34455120

[58] Tusiewicz K, Kuropka P, Workiewicz E, Wachełko O, Szpot P, Zawadzki M. 2023 Nitrites: an old poison or a current hazard? Epidemiology of intoxications covering the last 100 years and evaluation of analytical methods. Toxics **11**, 832. (10.3390/toxics11100832) 37888684 PMC10611400

[59] Liu RT, Xu ZL, Li FM, Chen FY, Yu JY, Yan Y, Chen Y, Xia BY. 2023 Recent advances in proton exchange membrane water electrolysis. Chem. Soc. Rev. **52**, 5652–5683. (10.1039/d2cs00681b) 37492961

[60] Lucas E, Martín AJ, Mitchell S, Nabera A, Santos LF, Pérez-Ramírez J, Guillén-Gosálbez G. 2024 The need to integrate mass- and energy-based metrics with life cycle impacts for sustainable chemicals manufacture. Green Chem. **26**, 9300–9309. (10.1039/d4gc00394b)

[61] Protsenko V. 2025 Supplementary from: Towards sustainable urea electrooxidation: a thermodynamic and green chemistry evaluation of alternative pathways. Dryad Digital Repository.. (10.5061/dryad.b2rbnzssv) PMC12289193 40708664

[62] Protsenko V. 2025 Supplementary material from: Toward sustainable urea electrooxidation: A thermodynamic and green chemistry evaluation of alternative pathways. FigShare. (10.6084/m9.figshare.c.7926418) PMC12289193 40708664

